# Assessing the relation between alcohol consumption and risk of disease and mortality

**DOI:** 10.1186/s12937-021-00714-4

**Published:** 2021-06-28

**Authors:** Benyamin Alam, Mehreen Anwar, Leonardo Bareli, Shamia Chowdhury

**Affiliations:** University of Manchester, Oxford Road, M13 9PL Manchester, UK

Dear editor,

As medical students at the University of Manchester, we read the article ‘Alcohol consumption and risk of cardiovascular disease, cancer and mortality: a prospective cohort study’ [1] published by the Nutrition Journal with great interest. Whilst it illustrates the negative impact that alcohol consumption can have on overall health, we believe that demographic confounding variables, limited assessment of consumption and insufficient follow up period detract from the validity of this study.

From reading the study, it is evident that a wide variety of cofounding variables have been accounted for. These include marital status, education level and income, however, one significant covariate was neglected. Ethnicity is a prominent confounding variable as it is known that the risk of CVD and cancers are affected by ones ethnicity and some ethnicities are at significantly greater risk than others [2]. Additionally distribution of ethnicities are likely to vary throughout the country with some regional lifestyles and diets having significant impact on health. Taking this into consideration, this confounding variable ought to have been eliminated by including a question about ethnicity in the questionnaires used.

Alcohol consumption was assessed using a self-administered questionnaire, which grouped different types of beverages into three distinct categories of ‘beer, wine, or hard liquor’. Further information that was collected included the amount and frequency ofintake. However, the recorded alcohol consumption values will be inaccurate as there can be great variance in the alcohol percengate [3]. To prevent this limitation, the study should have included the brand of the beverage, alongside the type, to give a more precise depiction of the individual’s alcohol consumption.

Participants medical records were reviewed 10 years following the initial questionnaires. This assesses the comparatively short-term impact of alcohol consumption. Alcohol related CVD, cancer and mortality normally develops insidiously and may take many years to manifest clinically, with an 86% incidence of CVD in patients of 80 years or older [4]. Therefore this study neglects the longer-term impacts of alcohol consumption on the participant’s health. To increase the clinical relevance of this study a further follow-up study should be held in the future in order to assess the negative health consequences of alcohol consumption later in life.

To conclude, this study articulates the wide variety of different health problems that alcohol consumption can cause. However, by increasing the demographic information ascertained, taking a more detailed assessment of alcohol consumption and scheduling an additional follow-up in the future, the clinical significance of this study could be greatly improved.

## Data Availability

Not applicable.
